# Hypoxia and the hypoxia inducible factor 1α activate protein kinase A by repressing RII beta subunit transcription

**DOI:** 10.1038/s41388-020-1223-6

**Published:** 2020-02-28

**Authors:** Kristin Lucia, Yonghe Wu, Jose Monteserin Garcia, Anne Barlier, Michael Buchfelder, Wolfgang Saeger, Ulrich Renner, Günter K. Stalla, Marily Theodoropoulou

**Affiliations:** 1 Department of Endocrinology, Max Planck Institute of Psychiatry, Munich, Germany; 2 Medizinische Klinik und Poliklinik IV, Ludwig-Maximilians-Universität München, Munich, Germany; 3 Division of Molecular Genetics, Deutsches Krebsforschungszentrum, Heidelberg, Germany; 4 Centre de Recherche en Neurobiologie et Neurophysiologie de Marseille, Marseille, France; 5 Department of Neurosurgery, Klinikum der Universität Erlangen, Erlangen, Germany; 6 Department of Neuropathology, Universität Hamburg, Hamburg, Germany; 7 Present Address: Department of Neurosurgery, Charité-Universitätsmedizin, Berlin, Germany; 8 Present Address: Division of Molecular Genetics, Deutsches Krebsforschungszentrum, Heidelberg, Germany

**Keywords:** Pituitary tumours, Mechanisms of disease

## Abstract

Overactivation of the cAMP signal transduction pathway plays a central role in the pathogenesis of endocrine tumors. Genetic aberrations leading to increased intracellular cAMP or directly affecting PKA subunit expression have been identified in inherited and sporadic endocrine tumors, but are rare indicating the presence of nongenomic pathological PKA activation. In the present study, we examined the impact of hypoxia on PKA activation using human growth hormone (GH)-secreting pituitary tumors as a model of an endocrine disease displaying PKA-CREB overactivation. We show that hypoxia activates PKA and enhances CREB transcriptional activity and subsequently GH oversecretion. This is due to a previously uncharacterized ability of HIF-1α to suppress the transcription of the PKA regulatory subunit 2B (*PRKAR2B*) by sequestering Sp1 from the *PRKAR2B* promoter. The present study reveals a novel mechanism through which the transcription factor HIF-1α transduces environmental signals directly onto PKA activity, without affecting intracellular cAMP concentrations. By identifying a point of interaction between the cellular microenvironment and intracellular enzyme activation, neoplastic, and nonneoplastic diseases involving overactivated PKA pathway may be more efficiently targeted.

## Introduction

The protein kinase A (PKA)-signaling cascade transduces physiological hormone mediated processes and its deregulation plays a central role in the pathogenesis of neoplastic as well as nonneoplastic diseases. The PKA tetrameric holoenzyme is composed of regulatory (R) and catalytic (C) subunit dimers. The regulatory subunits are each present in alpha (α) and beta (β) isoforms (RIα, RIIα, RIβ, and RIIβ) and their expression and tissue-specific balance are essential in shaping the specificity and degree of its activity [1, 2]. When in the R_2_C_2_ conformation, PKA is catalytically inactive. Following the binding of the second messenger 3′5′-cyclic adenosine monophosphate (cAMP) to the regulatory subunit dimer, a conformational change occurs which allows the active catalytic subunit to dissociate and phosphorylate serine/threonine residues of substrate proteins, like cAMP-responsive element (CRE) binding protein (CREB) [3–5]. PKA is deregulated in several human cancers and in particular in endocrine tumors such as thyroid cancer, adrenal tumors (Cushing’s syndrome, Carney complex), and GH-secreting pituitary tumors (acromegaly) as a result of altered expression of its subunits [6–8].

Acromegaly is an endocrine neoplastic condition caused by excessive GH secretion from tumors of the anterior pituitary gland [9]. Under physiological conditions hypothalamic stimuli trigger GH synthesis by activating the cAMP/PKA-signaling cascade and somatostatin analogs that inhibit this pathway are the mainstay pharmacological treatment for patients with acromegaly [10]. Therefore, the pathophysiology of these tumors is tightly linked to an overactivated PKA-signaling cascade that in 40% of cases is due to activating mutations of the guanine nucleotide binding protein (G protein), alpha stimulating activity polypeptide 1 (*GNAS1*) gene (gsp oncogene) encoding for the Gsα subunit [11]. Recent whole-genome and -exome sequencing studies in acromegalic patients did not reveal any novel recurrent somatic mutations, which could account for the PKA overactivation in more than half of GH-secreting pituitary tumor cases [12, 13]. In contrast, an intriguing pathological feature of these tumors is their decreased vascular density when compared with the nontumorous anterior pituitary tissue [14].

Decreased vascular density is frequently observed in solid tumors where it leads to relative tissue hypoxia [15]. The cellular response to tissue hypoxia involves the oxygen-dependent stabilization and activation of a family of transcription factors known as hypoxia inducible factors (HIFs). HIF-1α is a well characterized member of the HIF family and is upregulated in various cancers compared with nontumorous tissues [16, 17]. Under nonhypoxic conditions, the activity of oxygen-dependent prolyl hydroxylases marks HIF-1α for VHL mediated ubiquitination and subsequent proteasomal degradation [18]. When the cellular oxygen concentration drops to near 2% O_2_, prolyl-hydroxylase activity is inhibited and HIF-1α remains stabilized and translocates to the nucleus where it associates with cofactors and binds to its cognate DNA motif thereby initiating the transcription of target genes [19, 20]. HIF-1α directly regulates adaptive processes, which can confer a survival advantage to cells in a hypoxic tissue microenvironment such as metabolic reprogramming towards a glycolytic phenotype, pH regulation, and nutrient uptake [21–24].

While germline and somatic mutations in the genes encoding for PKA regulatory subunits have been characterized, they are infrequent and little is known about nongenomic effectors of aberrant PKA activity in human tumors [7, 8, 25]. The role of the tumor microenvironment in shaping the phenotype of AIP-mutation-positive somatotroph tumors recently been highlighted [26]. We therefore hypothesized that the decreased vascular density observed in acromegalic pituitary tumors may contribute to the activated PKA found in the absence of stimulating *gsp* mutations. Studies in tissues under hypoxia due to myocardial or cerebral infarction demonstrated high phosphorylation levels of the PKA substrate CREB, suggesting that low oxygen availability may indeed influence PKA activity [27, 28]. In addition, there is evidence that hypoxia affects the intracellular location of PKA and subsequently its function [25]. Accordingly, the aim of this study was to investigate the nongenomic effect of hypoxia on PKA activation using GH-secreting pituitary tumor cells as a model.

We observed that human acromegalic tumors, but not the normal pituitary gland, present with HIF-1α immunoreactivity (IR) indicative of hypoxic state. In primary human tumor cell cultures as well as immortalized pituitary tumor cells, hypoxia, and HIF-1α increased PKA activity and its downstream targets CREB and GH synthesis without affecting intracellular cAMP concentrations. We show that HIF-1α represses the transcription of the gene encoding for RIIβ (*PRKAR2B*) by interacting with Sp1 and sequestering it from the promoter. The selective overexpression of RIIβ abolished the effects of HIF-1α on PKA activation and its downstream targets, suggesting that the decreased *PRKAR2B* transcription under hypoxia is sufficient to increase PKA activity. Indeed, human acromegalic tumors showed reduced *PRKAR2B* transcript levels that were negatively correlated with HIF-1α protein. Altogether these data showcase the important role of HIF-1α on PKA regulation which not only has consequences in the pathogenesis of endocrine tumors and their response to treatment, but may also provide a basis for intervening in nonneoplastic diseases such cardiac disorders in which hypoxia and PKA activation also play a central role.

## Materials and methods

### Compounds and antibodies

The adenylate cyclase activator forskolin was obtained from Sigma (St. Louis, MO, USA). Primary antibodies used in this study were: CREB (Cell Signaling, Cat. 86B10—western Blot, ChIP), phospho-CREB Ser133 (Cell Signaling Cat. 87G3—western Blot) Anti-FLAG M2 (Sigma, Cat. F3165—western Blot), Anti-HIF-1α (Novus, Cat. NB100134—western Blot, Immunohistochemistry, Co-Immunoprecipitation, ChIP), Pit-1 (Santa Cruz Cat. Sc-442—western Blot, ChIP), PP1a (Santa Cruz Cat. Sc-7482—western Blot), Sp1 (Santa Cruz Cat Sc59X—western Blot, ChIP), and PKA IIB Reg—H90 (Santa Cruz Cat. sc-25424). Secondary Antibodies conjugated to HRP (rabbit and mouse) used for western blot were obtained from Cell Signaling. Biotinylated Rabbit IgG for immunohistochemistry was obtained from Vector (Cat. BA1000). Normal rabbit and mouse IgG were obtained from Santa Cruz and used as negative controls for co-immunoprecipitation and ChIP studies.

### Immunohistochemistry

The tissue processing and immunohistochemistry of 39 paraffin embedded human GH-secreting pituitary tumors was performed as previously described [29]. All samples were obtained from patients undergoing transsphenoidal tumor resection following signed and informed consent and after approval of the local ethics committee. Following staining, the IR of HIF-1α was scored according to the following scheme: 1 = 10–30% IR, 2 = 31–60% IR, and 3 = 61–100% IR. Nine nondiseased human pituitary glands were obtained from autopsy cases of sudden death with no evidence of endocrine diseases, taken 8–12 h postmortem. Normal pituitary samples were subjected to the same processing and scoring as described for tumor samples. The removal and use of pituitary tissue was approved by the ethics committee of the Max-Planck-Institute of Psychiatry and informed consent was received from the relatives of donors.

### Cell culture

The rat GH-secreting pituitary tumor cell line GH3 was obtained from ATCC and cultured for maintenance in DMEM (Gibco 41965) supplemented with 10% heat inactivated fetal calf serum (Gibco 10270), 100 U ml^−1^ penicillin/streptomycin (Biochrom) and 2 nM glutamine (Biochrom). Processing and culturing of primary human acromegalic tumors was performed as previously described [30].

### Hypoxia chamber

The hypoxic treatment of cells was performed in a modular hypoxia incubator chamber (StemCell Technologies, Cat. 27310). For hypoxic incubation, cells were placed in the center of the chamber which was sealed shut and connected via a flow meter (StemCell Technologies, Cat. 27311) to a gas tank containing 1% O_2_, 5% CO_2_, and 94% N_2._ The modular chamber was placed in a standard humidified incubator at 37° for 6–12 h. A normoxic control was placed in the same incubator outside of the hypoxia chamber. Control of hypoxia was performed either by western blot for HIF-1α protein expression or hypoxia responsive element (HRE)-luciferase assay.

### Western blot

Sample preparation of snap-frozen tissue (human normal anterior pituitary gland and acromegalic pituitary tumors) was performed as follows: tissue samples were homogenized in ice-cold RIPA buffer (50 mM Tris pH 8.0, 150 mM NaCl, 1% NP–40, 0.5% sodium deoxycholate and 0.1% SDS) using an Ultra-Turrax. GH3 cell lysates were in RIPA buffer and disrupted using a 20G insulin needle. Protein concentration was determined with the Bradford assay. Immunoblot was performed using 10–15 µg to total protein in sample buffer (Roti-Load 1, Roth). Signals were detected using ECL Clarity (Biorad).

### Co-immunoprecipitation

Co-immunoprecipitation experiments in GH3 cells were performed on the nuclear fractions. For fractionation cells were collected by careful scraping and pelleted by centrifugation. The cell pellet was carefully resuspended in hypotonic cell lysis buffer and incubated on ice for 15 min. Following centrifugation, the supernatant was decanted and cells were disrupted again in hypotonic lysis buffer using a 20G insulin syringe and centrifuged. The supernatant containing the cytoplasmic fraction was separated and 75 µl of cell extraction buffer was used to resuspend the pellet. A final centrifugation step was performed and the supernatant containing the nuclear fraction was separated to a clean tube and subjected to preclearing for 30 min at 4 °C using 10 µL Protein G Dynabeads per 10^6^ cells. The immunoprecipitation reaction was performed using Protein G Dynabeads coupled to the primary antibody in a separate reaction. In total, 10 µL of the antibody-bead complexes were given to 30 µL of precleared lysates. Following rotation overnight at 4 °C, immune complexes were washed and suspended in sample buffer (RotiLoad 1, Roth) to be immunoblotted as described above.

### Chromatin immunoprecipitation

GH3 cells were processed with the EZ ChIP chromatin immunoprecipitation kit (Upstate), using rabbit anti-Pit-1 (Santa Cruz), anti-CREB (Cell Signaling), anti-HIF-1α (Novus), and anti-Sp1 (Santa Cruz) antibodies. Normal rabbit and mouse IgGs were used as negative control for the respective immunoprecipitation reactions. Primers against the rat *Gh* promoter were previously described [31]. Primers against the rat *Pit-1* promoter were 5′-TGACGTCAAATAAAGTTTCTGTTTT-3′ and 5′-TGTTAACCCGAACTGTCTTTCTTAC-3′ (Eurofins MWG Operon), and primers against rat *Prkar2B* were 5′-CACCAATGTGGAGGCTGAAGT-3′ and 5′-GCAAATCCCACGCTTCTTTCT-3′.

### PP1 activity

Phosphatase activity was measured using the Ser/Thr phosphatase assay kit 1 (Upstate) according to the manufacturer’s instructions, at OD_630nm_.

### PKA activity assay

The PepTag Nonradioactive Protein Kinase Assay Kit (Promega, Madison, USA) was used according to the manufacturer’s instructions. Imaging of the phosphorylated peptide substrate was performed using a ChemiDoc MP (Bio-Rad) and densitometric quantification was performed using Bio-Rad ImageLab 4.1 software.

### Plasmid transfection, RNA interference, and reporter assays

GH3 cells were transfected using SuperFect (Qiagen) [32]. Single-interfering RNAs (siRNAs) were against rat *HIF-1α* (Santa Cruz Cat. sc-45919) and rat *Creb1* (Santa Cruz Cat. sc-72030) and nonspecific scramble control (Santa Cruz Cat. sc-37007). The following expression plasmids were used: M7 pdn-PKA-GFP (Randall Moon; Addgene plasmid # 16716), RSV CREB (Marc Montminy; Addgene plasmid # 22394), RSV CREB-M1 (Marc Montminy; Addgene plasmid # 22395), PRKAR2B (Origene, SKU SC125501), and pCMV-3xFLAG-HIF-1α (gift of Eduardo Arzt (Buenos Aires Argentina)). In vitro mutagenesis was performed to change the Arginine^30^ to Alanine in the pCMV-3xFLAG-HIF-1α plasmid (QuickChange II-Direct Mutagenesis Kit, Agilent Technologies), and constructs were verified by sequencing (Sequiserve, Vaterstetten Germany). Luciferase reporter constructs used in this study were as follows: HRE-luciferase, a gift from Navdeep Chandel (Addgene plasmid # 26731) contains three hypoxia response elements from the Pgk-1 gene upstream of firefly luciferase. The pA3GHluc GH-luciferase, a gift from A. Gutierrez-Hartmann, Denver USA, has the proximal (_593) rat GH promoter upstream to the luciferase gene. The pCRE-luc construct (Mercury pathway profiling system; Clontech Laboratories, Mountain View, CA, USA) has the CRE upstream to the TATA box of the herpes simplex virus thymidine kinase promoter and firefly luciferase. The reporter genes were cotransfected with RSV-βGal. Luciferase activity was measured using a TriStar and galactosidase activity (*o*-Nitrophenyl β-D-galactopyranoside) in an absorbance plate reader at 405 nm. Relative luciferase values (Luc/Gal) were applied to normalize for transfection efficiency.

### Radioimmunoassays

Rat GH was measured as previously described [33]. Human GH was measured using a commercial kit (DRG Diagnostics, Cat. RIA-0225). To correct for possible effects of hypoxic incubation and transfection on the proliferation of GH3 cells, cell proliferation was measured in parallel using the nonradioactive WST-1 assay (Roche) and used to normalized GH values. Rat intracellular cAMP was measured using a commercial kit (Perkin Elmer, Cat. NEK033).

### Quantitative real-time RT-PCR (qPCR)

Total RNA was extracted from cells and tissues with Trizol reagent (Life Technology) according to the manufacturer’s instructions. Total of 1 µg RNA was reverse transcribed using the QuantiTect Reverse Transcription Kit (Qiagen) according to the manufacturer’s instructions. Quantitative real-time PCR was performed on a capillary LightCycler (Roche) using the QuantiFast SYBR Green PCR Kit (Qiagen) at a final volume of 10 μl. Expression levels of the housekeeping genes (human β-actin and rat TFII-β) were used for normalization. In the case of human pituitary tumors, only those that had been screened by conventional PCR for the presence of normal pituitary tissue in the sample were included in the study as previously described [34].

### Gsp mutation analysis

DNA from human acromegalic pituitary tumors (snap frozen) was used for the analysis of the *gsp* mutation status as previously described [11].

### Statistical analysis

Statistical analysis was performed using SPSS (PASW Statistics) 18. Differences in human tissue samples were assessed using the nonparametric Mann–Whitney *U*-Test with *P* < 0.05 considered as significant in samples with similar variance. Differences in cell line experiments were assessed using the Student’s *t* test with *P* < 0.05 considered as significant. Analysis of patient data was performed using linear regression analysis and Pearson’s *rho* with *P* < 0.05. Experiments with full data/replicate values were included for statistical analysis.

## Results

### HIF-1α is overexpressed in GH-secreting pituitary tumors

**Fig. 1:**
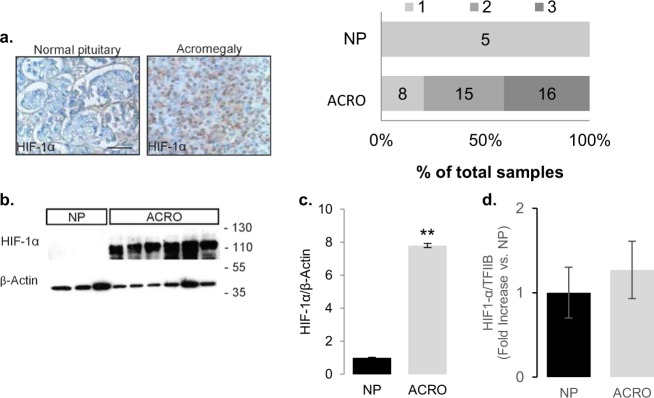
HIF-1 is upregulated in acromegalic pituitary tumors (ACRO) compared with the normal anterior pituitary gland (NP). **a** HIF-1α immunoreactivity in representative normal pituitary gland and acromegalic tumor. Signal is visualized with diaminobenzidine (DAB) staining (brown nuclei). Counterstaining with toluidine blue (blue nuclei). The graph shows the distribution of the HIF-1α immunoreactivity score on normal pituitary glands (*n* = 5) and acromegalic tumors (*n* = 39). The absolute numbers of samples are denoted in the graph bars. **b** Representative immunoblot for HIF-1α on normal pituitary glands (*n* = 3) and acromegalic tumors (*n* = 6). **c** Quantification of HIF-1α signal as determined by western blot on 5 normal pituitaries and 25 acromegalic tumors. Values are HIF-1α to β-actin ratio and presented as fold increase versus the mean normal pituitary values. Error bars: s.d. **P < 0.01 to normal pituitary glands (*U*-test). **d** Real-time RT-PCR data on RNA from the same samples as in **c**. Data are means ± standard deviation of two measurements and presented as HIF-1α/TFIIB fold increase versus the mean normal pituitary values *P < 0.05 (Student’s *t* test).

Immunohistochemistry performed on archival paraffin embedded GH-secreting pituitary tumors from patients with acromegaly and normal autoptic pituitary samples showed abundant nuclear HIF-1α staining in acromegalic tumors and virtually absent staining in the normal pituitary (Fig. 1a). These results were confirmed in western blot analysis which demonstrated strong expression in acromegalic tumors but absent HIF-1α signal in the normal pituitary (Fig. 1b, c). HIF-1α stability is mainly posttranscriptionally regulated, and while HIF-1α transcripts were detected in both normal pituitaries and acromegalic tumors, no significant differences were measured (Fig. 1d) indicating that the increased HIF-1α transcription per se is not responsible for the abundant protein expression observed in acromegalic tumors. As GH-secreting tumors carry oncogenic *gsp* mutations in 40% of cases [35], we examined the possible association between *gsp* status and HIF-1α protein levels in 21 acromegalic tumors (Table 1). Linear regression analysis showed no significant predictive value of the presence of the *gsp* oncogene on HIF-1α protein expression (Table 1). These data suggest that the increased HIF-1α expression does not occur secondary to *gsp* oncogenic mutations.

**Table 1:** Patient cohort used for mRNA and protein screening.

Case Nr.	Sex	Age	Grade	Gsp status	Gsa mRNA	HIF-1a protein
1	M	61	II	−	557	113
2	M	31	II	−	57	196
3	M	50	III	+	475	200
4	M	38	II	+	729	188
5	F	54	III	−	755	161
6	F	37	III	−	999	348
7	F	30	III	−	175	216
8	F	51	III	−	170	180
9	M	51	III	−	684	231
10	M	49	III	−	668	5
11	M	24	II	−	288	429
12	M	43	III	−	858	56
13	M	46	III	−	734	80
14	M	34	II	+	330	190
15	M	83	III	−	168	332
16	M	72	II	−	324	162
17	F	38	III	+	207	147
18	M	36	II	+	332	168
19	M	57	I	−	997	322
20	M	42	III	−	174	165
21	M	32	III	+	239	123

Patient cohort of the 21 acromegalic tumors that were analyzed by real-time PCR, western blot, and gsp mutational status. No significant predictive value of the presence of the *gsp* mutation status on HIF-1α protein expression was observed (F(1,20) = 0.479, *P* = 0.497, R^2^ = −0.025).

### HIF-1a mediates the effects of hypoxia on GH synthesis

**Fig. 2:**
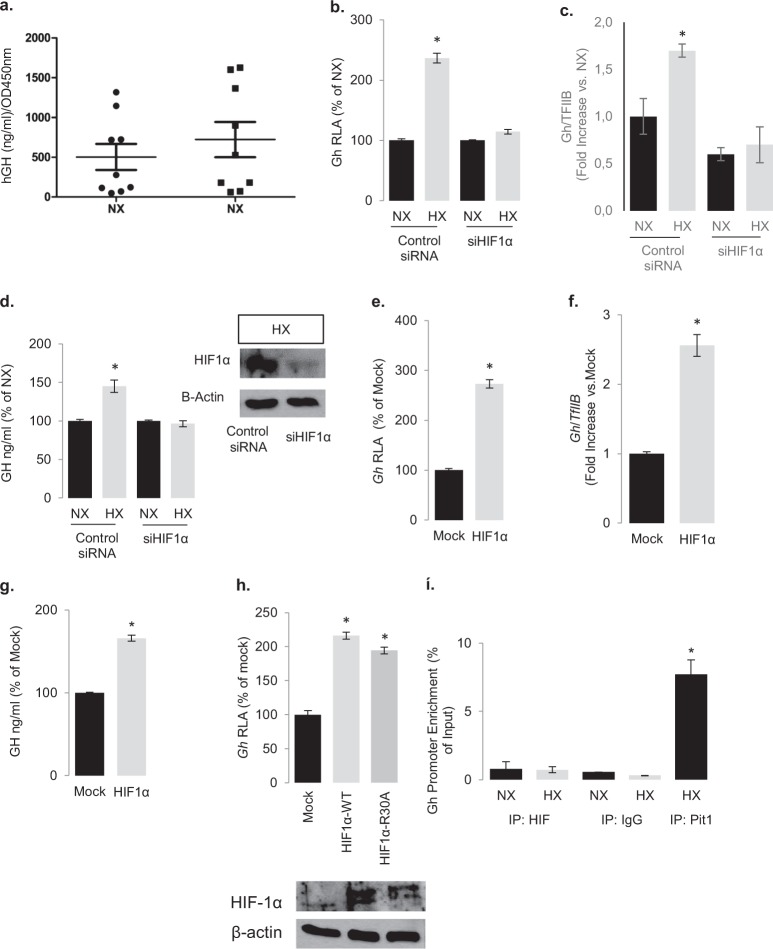
Hypoxia and HIF-1α increase GH synthesis. **a** Effect of hypoxia (1% O_2_ for 18 h) on GH secretion on nine human acromegalic tumors in primary cell culture. For all cell culture experiments, each GH RIA value was divided to respective cell viability count as determined by WST-1 at OD450nm. Every condition was in quadruplicates and data are means ± SEM. *P < 0.05 to normoxia (NX) (Student’s *t* test). **b** Effect of 18 h hypoxia on *Gh* promoter activity in GH3 cells transfected with the rat Gh-luc plasmid (pA3GHluc). Luc/βGal: luciferase to β-galactosidase ratio. Data are means ± SEM from three experiments and are presented as percentage of each normoxia (NX). *P < 0.05 to each normoxia (Student’s *t* test). RLA, relative luciferase activity. **c** Effect of hypoxia on endogenous rat Gh transcription as determined by real-time RT-PCR. Data are *Gh*/*TfIIb* from three independent experiments and presented as fold increase to each normoxia. *P < 0.05 (Student’s *t* test). **d** Effect of hypoxia on GH secretion. In **b**–**d** transfection with 100 nM HIF-1α siRNA for 48 h abolished the effect of hypoxia. The immunoblot shows as control the HIF-1α siRNA knockdown efficacy in hypoxic cells. Data are means ± SEM from three experiments each done in triplicates. *P < 0.05 to each normoxia (Student’s *t* test). Effect of HIF-1α overexpression on (**e**) rat *Gh* promoter activity, (**f**) endogenous rat *Gh* transcription, and (**g**) secretion. Data are presented as fold increase versus mock control (pCMV empty vector). *P < 0.05 to mock (Student’s *t* test). **h** Effect of the HIF-1α mutant HIF-1αR30A on rat *Gh* promoter activity. Data are means ± SEM from three experiments and are presented as percentage of mock transfected control. Inset shows immunoblot for HIF-1α in the overexpressing cells. **i** Chromatin immunoprecipitation showing absence of HIF-1α binding to the endogenous rat *Gh* promoter in GH3 cells under normoxia or hypoxia, in contrast to the positive control Pit-1 (shown in hypoxic cells). Rabbit IgG was used as a control.

To examine the impact of HIF-1α on PKA activity we first studied its effect on GH synthesis, which is tightly regulated by the PKA-signaling cascade [36–39] and is therefore a physiologically relevant readout of PKA activity. Primary cultures of human GH-secreting tumors incubated under hypoxia (1% O_2_ for 18 h) showed a significant increase in GH secretion compared with parallel normoxic cultures of the same tumor (Fig. 2a). Similarly, immortalized GH-secreting pituitary tumor GH3 cells incubated under hypoxic conditions showed increased rat *Gh* promoter activity, transcription and hormone secretion, and these effects were abrogated by knocking down HIF-1α with RNA-interference (Fig. 2b–d). Transient overexpression of HIF-1α also significantly increased *Gh* promoter activity as well as transcription and secretion (Fig. 2e–g). An HIF-1α construct unable to bind to the consensus HRE due to a single amino acid mutation in its DNA-binding domain (HIF^R30A^) [40] also increased *Gh* promoter activity to a similar extent as the wild-type HIF-1α (Fig. 2h, Supplementary Figs. 1 and 2), indicating that HIF-1a was not directly promoting growth hormone transcription via promoter binding. In addition, chromatin immunoprecipitation showed no enrichment of HIF-1α on the *Gh* promoter in contrast to the abundant binding of Pit-1 which was used as positive control [41] (Fig. 2i). These results effectively rule out a direct DNA binding as the cause of HIF-1α–induced GH synthesis and point to an integration point upstream to its transcription.

### HIF-1α affects CREB phosphorylation and transcriptional activity

**Fig. 3:**
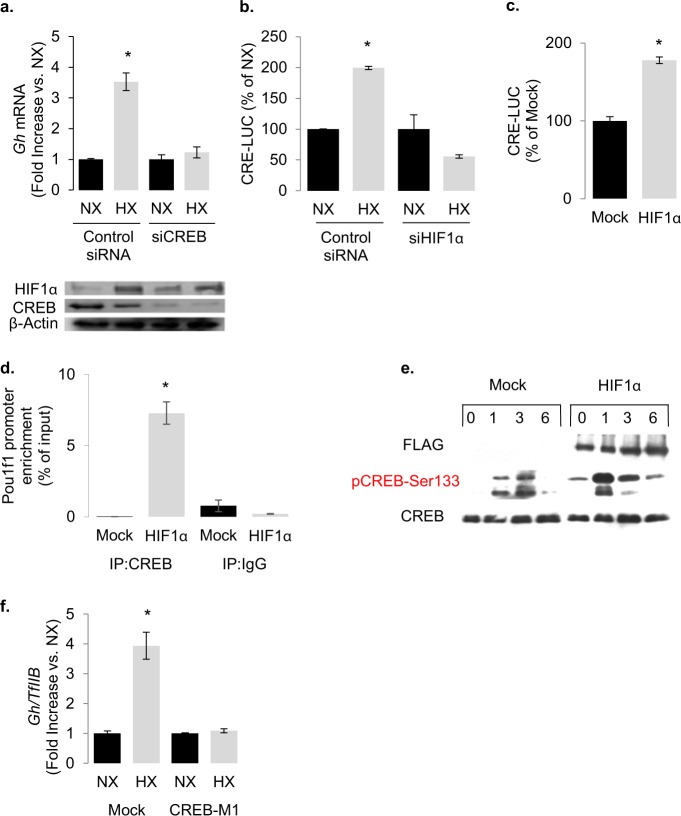
Hypoxia and HIF-1 trigger CREB activity. **a** Knocking down CREB with siRNA abolishes the effect of hypoxia on endogenous rat *Gh* transcription in GH3 cells as determined by real-time RT-PCR. Immunoblot shows the knockdown efficacy of the CREB siRNA (**b**) effect of hypoxia (1% O_2_ for 18 h) on CRE induced luciferase activity. Transfection with 100 nM HIF-1α siRNA for 48 h abolished the effect of hypoxia. Luc/βGal: luciferase: β-galactosidase ratio. Data are means ± SEM of three experiments and expressed as percentage of each normoxia control. *P < 0.05 (Student’s *t* test). **c** Effect of HIF-1α overexpression on CRE luciferase activity. Data are means ± SEM of three experiments and expressed as percentage of mock control. *P < 0.05 (Student’s *t* test). **d** Chromatin immunoprecipitation showing increased CREB binding to the endogenous rat Pou1f1 (encoding for Pit-1) promoter in GH3 cells overexpressing HIF-1α. Rabbit IgG was used as a control. Data are arbitrary units from two independent experiments, presented as% of input. **P < 0.01 (Student’s *t* test). **e** Immunoblot showing that HIF-1α overexpression increases basal and forskolin (5 µM, 1–6 h)-induced pCREB-Ser133 levels. It also shows that forskolin-induced pCREB-Ser133 remains elevated in HIF-1α overexpressing GH3 cells, while it is back to basal after 6 h in the mock plasmid control transfected cells. **f** Hypoxia fails to increase rat *Gh* transcription in GH cells overexpressing CREB-M1 (CREBS133A) a mutant that cannot be phosphorylated by PKA. Data are *Gh/TfIIb* and presented as fold increase to each normoxia (NX). *P < 0.05 to each normoxia (Student’s *t* test).

Pituitary GH transcription is stimulated by CREB both directly, through the binding to its canonical CRE promoter sequences, and indirectly by promoting the transcription of the *POU1F1* gene, which encodes for Pit-1 [36, 41, 42]. Indeed, CREB inhibition with RNA interference abrogated the ability of hypoxia to increase rat *Gh* transcription (Fig. 3a). Hypoxia significantly increased CRE transcriptional activity in a HIF-1α-dependent manner (Fig. 3b), and HIF-1α overexpression both triggered CRE transcriptional activity (Fig. 3c) and increased the recruitment of CREB to endogenous *Pou1f1* promoter in GH3 cells (Fig. 3d). A prerequisite for CREB DNA binding and transcriptional activity is the phosphorylation of its Ser^133^ residue by PKA [43]. The phosphorylation pattern of CREB displays a burst-attenuation kinetic [44] with maximal phosphorylation occurring at ~1 h, followed by sequentially decreasing phosphorylation due to the action of the serine/threonine protein phosphatase PP1, which returns to basal levels at ~6 h [45–47]. HIF-1α overexpression increased basal phosphorylation of CREB and blunted the physiological attenuation of forskolin-induced Ser^133^ phosphorylation over 6 h (Fig. 3e, Supplementary Fig. 3). Ser^133^ phosphorylation of CREB was also found under hypoxic incubation (Supplementary Fig. 4). No changes in PP1 protein expression or phosphatase activity were observed, indicating that HIF-1α does not block CREB dephosphorylation (Supplementary Fig. 5). In contrast, overexpression of a CREB^S133A^ mutant (CREB-M1) that cannot be phosphorylated [43] blunted the effects of hypoxia on endogenous rat *Gh* transcription in GH3 cells (Fig. 3f, Supplementary Fig. 6), indicating the importance of CREB phosphorylation in hypoxia’s action.

### HIF-1α and hypoxia activate PKA

**Fig. 4:**
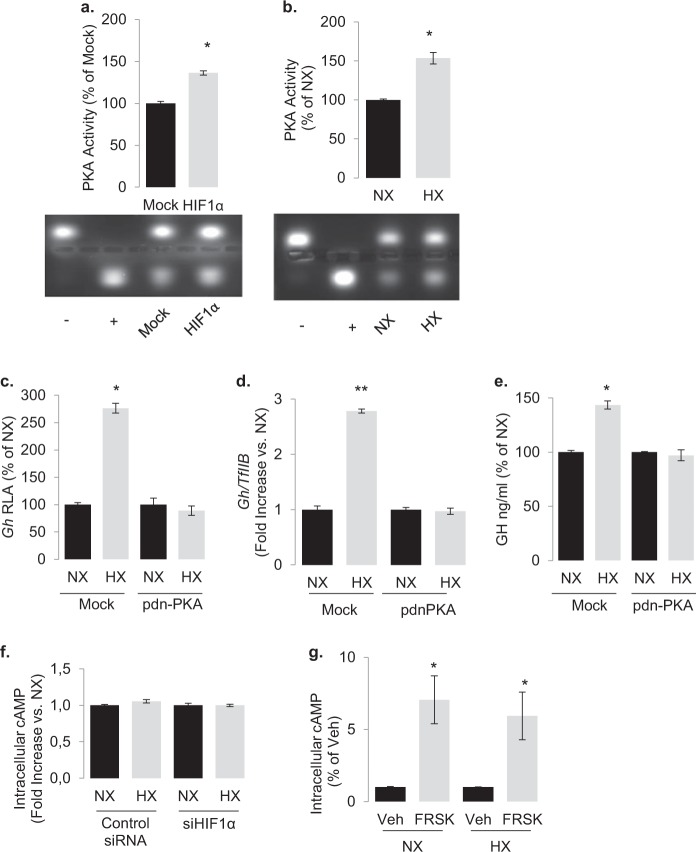
Hypoxia and HIF-1α stimulate PKA activity. The effect of (**a**) HIF-1α overexpression and (**b**) hypoxia on PKA activity as determined with a nonradioactive commercial kit (Promega). Blots show imaging of the phosphorylated peptide substrate. Data are arbitrary units presented as % of mock (empty pCMV plasmid) control or normoxia (NX). *P = 0.040 to mock and *P* = 0.019 to normoxia (Student’s *t* test). Overexpression of a dominant negative catalytically inactive PKA (pdn-PKA) abolishes the stimulatory action of hypoxia on (**c**) *Gh* promoter activity, (**d**) endogenous rat *Gh* transcription, and (**e**) GH secretion. Data are means ± SEM from two experiments and presented as percentage of fold increase to each normoxia (NX). *P < 0.05 to each normoxia (Student’s *t* test). Hypoxia has no effect on basal (**f**) and forskolin-induced (**g**) cAMP levels. Data are means ± SEM of two experiments. **P < 0.001 to vehicle (Student’s *t* test).

PKA is the major kinase phosphorylating CREB [48, 49]. Both HIF-1α overexpression and hypoxic incubation significantly increased PKA activity (Fig. 4a, b). Overexpression of a dominant negative catalytically inactive PKA in GH3 cells abolished the stimulatory action of hypoxia on GH promoter activity, transcription, and secretion (Fig. 4c–e), demonstrating that hypoxia and consequently HIF-1α requires a catalytically active PKA to mediate its effects. PKA activation first occurs following an increase in intracellular cAMP concentrations [48, 49]. However, neither HIF-1α inhibition with RNA interference nor hypoxic incubation affected basal and forskolin-induced intracellular cAMP levels (Fig. 4f, g), indicating that HIF-1α activates PKA independently of cAMP.

### HIF-1α suppresses Prkar2b transcription

**Fig. 5:**
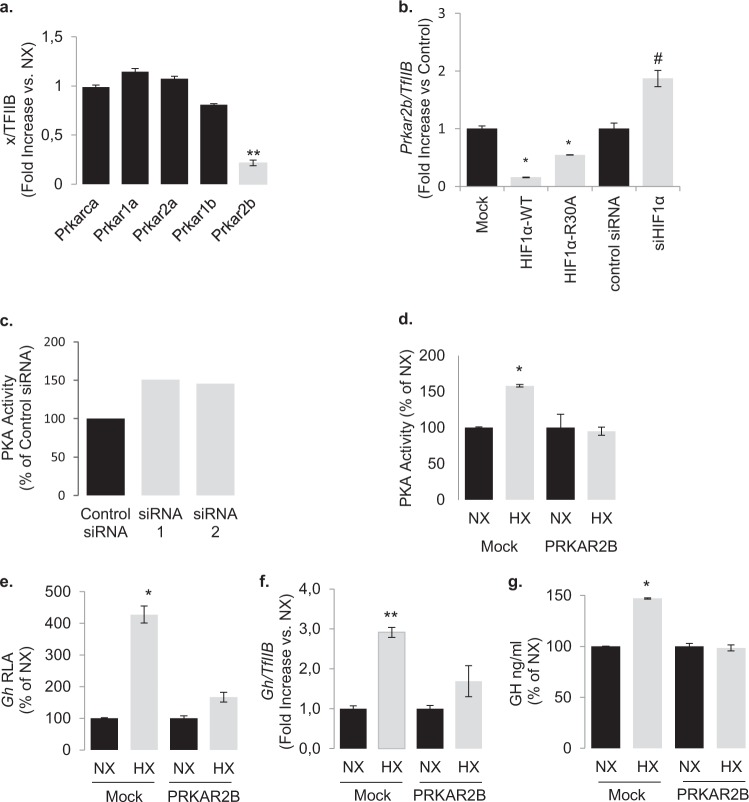
Hypoxia and HIF-1α downregulates the transcription of gene encoding for the PKA regulatory subunit RIIB. **a** Expression of the genes encoding for PKA regulatory and catalytic subunits in GH3 cells grown under hypoxic conditions (1% O_2_ for 18 h) as determined by real-time RT-PCR. Data are <*gene*>/*TfIIb*, means ± SEM from two experiments and presented as fold change to each normoxia. **P < 0.01 to each normoxia (NX) (Student’s *t* test). **b** Effect of HIF-1α overexpression or knockdown with siRNA on *Prkar2b* transcription. *P < 0.05 to pCMV empty vector, ^#^*P* < 0.05 to scrambled siRNA control (Student’s *t* test). **c** Two siRNA constructs targeting *Prkar2b* in GH3 cells caused an increase in PKA activity. Data are means representative of the two individual constructs and expressed as % of control siRNA. Hypoxia (1% O_2_ for 18 h) has no effect on (**d**) PKA activity, (**e**) rat *Gh* promoter activity, (**f**) endogenous rat *Gh* transcription, and (**g**) GH secretion in GH3 cells overexpressing the regulatory subunit RIIB. Data are means ± SEM from two experiments. *P < 0.05 (Student’s *t* test).

PKA activity can be dysregulated as a result of altered subunit expression patterns activity [48, 49]. As a transcriptional regulator, we therefore speculated that HIF-1α may induce basal PKA activity by altering the balance of the expression of the different PKA subunits. HIF-1α overexpression and incubation under hypoxic conditions significantly suppressed the expression of the gene encoding for RIIβ (*Prkar2b*) and did not significantly affect the expression of the genes encoding for the other regulatory subunits (*Pkar1a*, *Prkar1b*, and *Prkar2a*) and the catalytic subunit (*Prkaca*) (Fig. 5a). Similarly, overexpression of both wild type and the HIF-1α^R30A^ suppressed *Prkar2b* transcription, whereas HIF-1α inhibition using RNA interference increased *Prkar2b* gene transcription (Fig. 5b). To determine whether the decreased expression of the *Prkar2b* gene impacts the catalytic activity of the PKA enzyme we reduced its expression with three different siRNA constructs and observed increase in PKA activity (Fig. 5c). In contrast, overexpression of the regulatory RIIβ subunit compromised the stimulatory action of hypoxia on PKA activity and blunted its effects on GH synthesis (Fig. 5d–g).

**Fig. 6:**
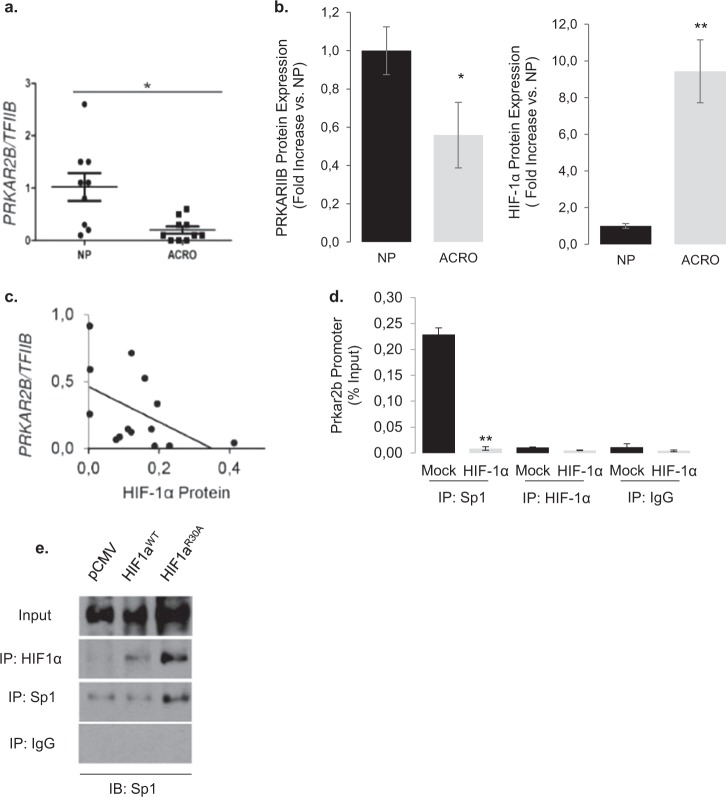
HIF-1a modulates PRKAR2B expression via Sp1 promoter binding and their expression levels are inverse correlated in acromegalic tumors. **a**, **d** Chromatin immunoprecipitation showing decreased Sp1 binding on the *Prkar2b* promoter in GH3 cells overexpressing HIF-1α. No DNA binding was quantified with HIF-1α. Rabbit IgG was used as control. **b**, **e** Co-immunoprecipitation experiment showing that both HIF-1α and HIF-1αR30A physically associate with Sp1. HIF-1α and Sp1 immunoprecipitates were blotted for Sp1. Proteins immunoprecipitated with rabbit IgG were used as controls. **c**, **a** Expression of *PRKAR2B* gene in normal pituitary glands (*n* = 9) and acromegalic tumors (ACRO, *n* = 10). Data are *PRAKR2B*/*TFIIB*. *P = 0.006 (*U*-test). **d**, **b** Western blot analysis of normal pituitary (NP) and GH-secreting tumors (ACRO) showed a significant loss of PRKAR2B protein expression, whereas HIF-1α protein levels measured in the same tumors are significantly increased in ACRO vs NP (*n* = 4 per group due to scarcity of material for dual measurement). **e**, **c** Linear regression analysis of *PRKAR2B* transcript and HIF-1α protein levels in 14 acromegalic tumors. *PRKAR2B* was determined by real-time RT-PCR and values are *PRKAR2B*/*TFIIB* arbitrary units. HIF-1α protein was quantified from immunoblots (Chemidoc). Kendall’s τ = −0.663, *r*_t_ = −0.480, *P* = 0.009.

These observations suggest that hypoxia via HIF-1α may render PKA active by suppressing *Prkar2b* transcription. *Prkar2b* gene transcription is regulated by the transcription factor Sp1 that binds on the GC-rich sequences of its promoter [50, 51]. As HIF-1α is able to bind and sequester Sp1 [52], we investigated whether it may physically interact with and sequester it away from the endogenous *Prkar2b* promoter in GH3 cells. Indeed, chromatin immunoprecipitation experiments revealed that GH3 cells overexpressing HIF-1α showed decreased Sp1 enrichment to the *Prkar2b* promoter compared with IgG controls (Fig. 6a, d). No promoter enrichment of HIF-1α on *Prkar2b* was observed confirming that it does not affect *Prkar2b* transcription by direct DNA binding. Hypoxia, HIF-1α silencing, and transient overexpression of HIF-1α did not affect Sp1 transcription (Supplementary Fig. 7). However, HIF-1α co-immunoprecipitated with Sp1 in hypoxic GH3 cells confirming their physical interaction in pituitary tumor cells (Fig. 6b, e).

This observation suggests that high levels of HIF-1α may compromise *PRKAR2B* transcription. Indeed in GH-secreting pituitary tumors from patients with acromegaly (*n* = 10), we confirmed a significant decrease in *PRKAR2B* expression (Fig. 6a) and a significant increase in HIF-1a expression compared with normal anterior pituitary glands (Fig. 6c, a) and this was also shown at protein level (Fig. 6d, b). In contrast, no significant changes were observed in the expression of the genes encoding for all the other subunits (Supplementary Fig. 8). In addition, we observed a significant negative correlation between HIF-1α protein and *PRKAR2B* mRNA levels in the GH-secreting pituitary tumors (Fig. 6e, c).

Altogether, these data demonstrate that hypoxia and HIF-1α ensure PKA activation by downregulating RIIβ, and this has physiological consequences on endocrine cells undergoing hypoxia during tumorigenesis.

## Discussion

In cancer, hypoxia has long been recognized as a contributing factor to the development and survival of tumor cells [53], and HIF-1α has been identified as a key mediator of the adaptive processes which confer a survival advantage in the hypoxic tumor microenvironment [21, 22, 54]. Our findings delineate a novel pathogenic mechanism through which HIF-1a can amplify PKA signaling independently of *gsp* mutations or intracellular cAMP concentrations. In GH-secreting tumors, whose pathophysiology is closely linked to PKA activity, we show that HIF-1α suppresses the transcription of the gene that encodes for RIIβ thereby promoting PKA activation, which in turn leads to greater activation of downstream targets such as CREB and ultimately promotes excessive GH synthesis.

There has been previous evidence that hypoxia and HIF-1α may affect PKA activity. First, in a model of melanoma it was demonstrated that the PKA scaffold protein AKAP12v2 is a direct transcriptional target of HIF-1α, and its hypoxic induction effectively enhances the migratory capacity of melanoma cells [25]. At the organelle level, hypoxia can promote the ubiquitination and degradation of the mitochondrial AKAP121, therefore attenuating PKA-CREB signal transduction to the outer mitochondrial membrane during brain ischemia [55]. Finally, in A549 lung cancer cells it was shown that long-term (2 days) hypoxic incubation can increase the relative expression of *PRKACA*, indicating that this may occur independently of HIF-1α transcriptional regulation [56].

In our system, both hypoxia and HIF-1α overexpression did not affect *PRKACA* expression, but dramatically repressed the transcription of *PRKAR2B*. Furthermore, this repression was essential for the stimulatory action of hypoxia and HIF-1a on the downstream PKA read-outs CREB and GH synthesis, providing with novel mechanistic insights into the nature of HIF-1α–PKA interactions. In fact, we found that the stimulatory effect of HIF-1α on PKA activity in GH-secreting pituitary tumor cells occurs specifically through suppression of RIIβ. In contrast to many other protein kinases which are regulated by the turnover of an activation loop phosphate, PKA is regulated by the composition of its holoenzyme structure, which determines its activation potential by cAMP [57]. As such, alterations in the structure of the holoenzyme complex make it susceptible to deregulation of kinase activity [48, 49]. As we found no significant alterations in intracellular cAMP levels, we propose that the suppression of *PRKAR2B* expression is the sole trigger of overactive PKA under these conditions. Therefore, *PRKAR2B* poses to be the first direct target of HIF-1α within the PKA holoenzyme.

Previous studies have shown that loss of RIα/β is associated with more aggressive tumor behavior [49, 58, 59], while the expression of RIIα/β promotes cell cycle arrest [52, 60]. In this sense, our findings that suppression of RIIβ in pituitary tumor cells leads to increased PKA activity and GH hypersecretion are in line with these previous findings. However, as RIα/β and RIIα/β are not only functionally nonredundant but also display a tissue-specific pattern of distribution [57], we believe the effects of loss or gain of a specific isoform should not be generalized without biological validation in the particular system of interest.

To date, the regulatory Iα subunit has been of particular interest in the pituitary as mutations mapping to its locus at 17q22-24 are commonly found in patients with Carney Complex, a multiple neoplasia syndrome which is associated with abnormal GH and prolactin secretion [7]. Large-scale genetic screening of sporadic acromegalic cases have revealed no loss of *PRKAR1A* gene expression indicating that *PRKAR1A* mutations are not involved in GH hypersecretion outside of the Carney Complex [12, 13, 50]. Given the paucity of evidence supporting mutations of the PKA subunits as being causative of GH hypersecretion in sporadic cases, our study presents a novel nongenomic mechanism through which HIF-1α acts as a surrogate of the tumor microenvironment to influence GH synthesis.

We found that HIF-1α downregulates *PRKAR2B* transcription but we did not detect any direct binding to its promoter. While HIF-1α can directly activate the transcription of a multitude of target genes via its constitutive DNA binding domain (CGTC), its role in transcriptional repression remains less well characterized. Genome-wide association studies of HIF-1α DNA binding and transcription profiling have shown that HIF-1α-dependent gene suppression most commonly occurs through indirect mechanisms such as the HIF-1α upregulates the transcription of transcriptional repressors that then suppress the expression of the affected genes [51, 61]. A less characterized mechanism is through physical interaction and sequestration of transcription factors from their target promoter sequence. In this context, HIF-1α was shown to compete with Sp1 for c-Myc, resulting in the transcriptional repression of the Myc target gene MutSα [62]. The *PRKAR2B* promoter has CG-rich regions with Sp1-binding sites [63, 64]. We found that HIF-1α physically interacts with and decreases Sp1 binding from the *Prkar2b* promoter therefore supporting the concept of HIF-1α-mediated transcriptional repression beyond the HRE-dependent regulation of gene expression. This effect of HIF-1a occurs solely by sequestering Sp1 and does not affect total Sp1 levels. These results point to a general mechanism through which HIF-1α may suppress the transcription of genes with CG-rich promoters.

A considerable amount of work has described the regulation action of PKA on HIF-1a [65]. Upstream PKA activators modulate HIF-1α transcriptional activation of target genes such as VEGF-A in lung cancer models [66]. In fact, PKA directly phosphorylates HIF-1α in endothelial cells under intermittent hypoxia [67]. Indeed, two putative PKA phosphorylation sites on HIF-1a Thr^63^ and Ser^692^ promote its stabilization independently of prolyl hydroxylation in rat cardiomyocytes [68]. This shifted the focus on the role of PKA-mediated regulation of HIF in models of congestive heart failure, as PKA plays an important role for signal transduction through β-adrenergic receptors in cardiomyocytes. Furthermore, inhibition of β-adrenergic receptors reduces the hypoxia-induced stabilization of HIF-1α in primary human endothelial cells [69]. Our study demonstrates that the PKA–HIF-1α interaction is reciprocal, with HIF-1α exerting a positive feedback on PKA.

Taken together with our findings of a novel mechanism of PKA activation through HIF-1a, the possibility of a feed-forward loop between HIF-1α and PKA may be of significant interest in regards to examining nongenomic mechanisms of PKA activation in both neoplastic and nonneoplastic diseases. Our studies in GH-secreting pituitary tumor cells provide with a new link between an environmental stressor in the form of hypoxia and an important intracellular physiological regulator of pituitary function. Given the diversity of cellular processes which are coordinated by PKA [70, 71], these observations may have implications reaching beyond endocrine pathology.

## Supplementary information

Supplementary figures

## References

[1] Amieux PS, Cummings DE, Motamed K, Brandon EP, Wailes LA, Le K (1997). Compensatory regulation of RIalpha protein levels in protein kinase A mutant mice. J Biol Chem.

[2] Brandon EP, Logue SF, Adams MR, Qi M, Sullivan SP, Matsumoto AM (1998). Defective motor behavior and neural gene expression in RIIbeta-protein kinase A mutant mice. J Neurosci.

[3] Mellon PL, Clegg CH, Correll LA, McKnight GS (1989). Regulation of transcription by cyclic AMP-dependent protein kinase. Proc Natl Acad Sci USA.

[4] Adams JA, Taylor SS (1992). Energetic limits of phosphotransfer in the catalytic subunit of cAMP-dependent protein kinase as measured by viscosity experiments. Biochemistry..

[5] Kim C, Cheng CY, Saldanha SA, Taylor SS (2007). PKA-I holoenzyme structure reveals a mechanism for cAMP-dependent activation. Cell..

[6] Sandrini F, Matyakhina L, Sarlis NJ, Kirschner LS, Farmakidis C, Gimm O (2002). Regulatory subunit type I-alpha of protein kinase A (PRKAR1A): a tumor-suppressor gene for sporadic thyroid cancer. Genes Chromosomes Cancer.

[7] Kirschner LS, Carney JA, Pack SD, Taymans SE, Giatzakis C, Cho YS (2000). Mutations of the gene encoding the protein kinase A type I-alpha regulatory subunit in patients with the Carney complex. Nat Genet.

[8] Beuschlein F, Fassnacht M, Assié G, Calebiro D, Stratakis CA, Osswald A (2014). Constitutive activation of PKA catalytic subunit in adrenal Cushing’s syndrome. N Engl J Med.

[9] Melmed S (2009). Acromegaly pathogenesis and treatment. J Clin Investig.

[10] Katznelson L, Laws ER, Melmed S, Molitch ME, Murad MH, Utz A (2014). Acromegaly: an endocrine society clinical practice guideline. J Clin Endocrinol Metab.

[11] Barlier A, Gunz G, Zamora AJ, Morange-Ramos I, Figarella-Branger D, Dufour H (1998). Pronostic and therapeutic consequences of Gs alpha mutations in somatotroph adenomas. J Clin Endocrinol Metab.

[12] Välimäki N, Demir H, Pitkänen E, Kaasinen E, Karppinen A, Kivipelto L (2015). Whole-genome sequencing of growth hormone (GH)-secreting pituitary adenomas. J Clin Endocrinol Metab.

[13] Ronchi CL, Peverelli E, Herterich S, Weigand I, Mantovani G, Schwarzmayr T (2016). Landscape of somatic mutations in sporadic GH-secreting pituitary adenomas. Eur J Endocrinol.

[14] Turner HE, Nagy Z, Gatter KC, Esiri MM, Harris AL, Wass JA (2000). Angiogenesis in pituitary adenomas and the normal pituitary gland. J Clin Endocrinol Metab.

[15] Wilson WR, Hay MP (2011). Targeting hypoxia in cancer therapy. Nat Rev Cancer.

[16] Zhong H, De Marzo AM, Laughner E, Lim M, Hilton DA, Zagzag D (1999). Overexpression of hypoxia-inducible factor 1alpha in common human cancers and their metastases. Cancer Res.

[17] Talks KL, Turley H, Gatter KC, Maxwell PH, Pugh CW, Ratcliffe PJ (2000). The expression and distribution of the hypoxia-inducible factors HIF-1alpha and HIF-2alpha in normal human tissues, cancers, and tumor-associated macrophages. Am J Pathol.

[18] Epstein AC, Gleadle JM, McNeill LA, Hewitson KS, O’Rourke J, Mole DR (2001). C. elegans EGL-9 and mammalian homologs define a family of dioxygenases that regulate HIF by prolyl hydroxylation. Cell..

[19] Semenza GL (2003). Targeting HIF-1 for cancer therapy. Nat Rev Cancer.

[20] Wang GL, Jiang BH, Rue EA, Semenza GL (1995). Hypoxia-inducible factor 1 is a basic-helix-loop-helix-PAS heterodimer regulated by cellular O2 tension. Proc Natl Acad Sci USA.

[21] Semenza GL (2013). HIF-1 mediates metabolic responses to intratumoral hypoxia and oncogenic mutations. J Clin Invest.

[22] Harris AL (2002). Hypoxia-a key regulatory factor in tumour growth. Nat Rev Cancer.

[23] Seagroves TN, Ryan HE, Lu H, Wouters BG, Knapp M, Thibault P (2001). Transcription factor HIF-1 is a necessary mediator of the pasteur effect in mammalian cells. Mol Cell Biol.

[24] Ryan HE, Poloni M, McNulty W, Elson D, Gassmann M, Arbeit JM (2000). Hypoxia-inducible factor-1alpha is a positive factor in solid tumor growth. Cancer Res.

[25] Finger EC, Castellini L, Rankin EB, Vilalta M, Krieg AJ, Jiang D (2015). Hypoxic induction of AKAP12 variant 2 shifts PKA-mediated protein phosphorylation to enhance migration and metastasis of melanoma cells. Proc Natl Acad Sci USA.

[26] Barry S, Carlsen E, Marques P, Stiles CE, Gadaleta E, Berney DM (2019). Tumor microenvironment defines the invasive phenotype of AIP-mutation-positive pituitary tumors. Oncogene..

[27] Prabu SK, Anandatheerthavarada HK, Raza H, Srinivasan S, Spear JF, Avadhani NG (2006). Protein kinase A-mediated phosphorylation modulates cytochrome c oxidase function and augments hypoxia and myocardial ischemia-related injury. J Biol Chem.

[28] Kitagawa K (2007). CREB and cAMP response element-mediated gene expression in the ischemic brain. FEBS J.

[29] Theodoropoulou M, Cavallari I, Barzon L, D’Agostino DM, Ferro T, Arzberger T (2004). Differential expression of menin in sporadic pituitary adenomas. Endocr Relat Cancer.

[30] Stalla GK, Stalla J, von Werder K, Müller OA, Gerzer R, Höllt V (1989). Nitroimidazole derivatives inhibit anterior pituitary cell function apparently by a direct effect on the catalytic subunit of the adenylate cyclase holoenzyme. Endocrinology..

[31] Ezzat S, Yu S, Asa SL (2005). The zinc finger Ikaros transcription factor regulates pituitary growth hormone and prolactin gene expression through distinct effects on chromatin accessibility. Mol Endocrinol.

[32] Theodoropoulou M, Zhang J, Laupheimer S, Paez-Pereda M, Erneux C, Florio T (2006). Octreotide, a somatostatin analogue, mediates its antiproliferative action in pituitary tumor cells by altering phosphatidylinositol 3-kinase signaling and inducing Zac1 expression. Cancer Res.

[33] Arzt E, Buric R, Stelzer G, Stalla J, Sauer J, Renner U (1993). Interleukin involvement in anterior pituitary cell growth regulation: effects of IL-2 and IL-6. Endocrinology.

[34] Pagotto U, Arzberger T, Theodoropoulou M, Grübler Y, Pantaloni C, Saeger W (2000). The expression of the antiproliferative gene ZAC is lost or highly reduced in nonfunctioning pituitary adenomas. Cancer Res.

[35] Hayward BE, Barlier A, Korbonits M, Grossman AB, Jacquet P, Enjalbert A (2001). Imprinting of the G(s)alpha gene GNAS1 in the pathogenesis of acromegaly. J Clin Investig.

[36] McCormick A, Brady H, Theill LE, Karin M (1990). Regulation of the pituitary-specific homeobox gene GHF1 by cell-autonomous and environmental cues. Nature..

[37] Struthers RS, Vale WW, Arias C, Sawchenko PE, Montminy MR (1991). Somatotroph hypoplasia and dwarfism in transgenic mice expressing a non-phosphorylatable CREB mutant. Nature..

[38] Bertherat J, Chanson P, Montminy M (1995). The cyclic adenosine 3’,5’-monophosphate-responsive factor CREB is constitutively activated in human somatotroph adenomas. Mol Endocrinol.

[39] Vitali E, Peverelli E, Giardino E, Locatelli M, Lasio GB, Beck-Peccoz P (2014). Cyclic adenosine 3’-5’-monophosphate (cAMP) exerts proliferative and anti-proliferative effects in pituitary cells of different types by activating both cAMP-dependent protein kinase A (PKA) and exchange proteins directly activated by cAMP (Epac). Mol Cell Endocrinol.

[40] Michel G, Minet E, Mottet D, Remacle J, Michiels C (2002). Site-directed mutagenesis studies of the hypoxia-inducible factor-1alpha DNA-binding domain. Biochim Biophys Acta.

[41] Ingraham HA, Chen RP, Mangalam HJ, Elsholtz HP, Flynn SE, Lin CR (1988). A tissue-specific transcription factor containing a homeodomain specifies a pituitary phenotype. Cell..

[42] Bodner M, Castrillo JL, Theill LE, Deerinck T, Ellisman M, Karin M (1988). The pituitary-specific transcription factor GHF-1 is a homeobox-containing protein. Cell.

[43] Gonzalez GA, Montminy MR (1989). Cyclic AMP stimulates somatostatin gene transcription by phosphorylation of CREB at serine 133. Cell..

[44] Hagiwara M, Alberts A, Brindle P, Meinkoth J, Feramisco J, Deng T (1992). Transcriptional attenuation following cAMP induction requires PP-1-mediated dephosphorylation of CREB. Cell..

[45] Canettieri G, Morantte I, Guzmán E, Asahara H, Herzig S, Anderson SD (2003). Attenuation of a phosphorylation-dependent activator by an HDAC-PP1 complex. Nat Struct Biol.

[46] Michael LF, Asahara H, Shulman AI, Kraus WL, Montminy M (2000). The phosphorylation status of a cyclic AMP-responsive activator is modulated via a chromatin-dependent mechanism. Mol Cell Biol.

[47] Yamamoto KK, Gonzalez GA, Biggs WH, Montminy MR (1988). Phosphorylation-induced binding and transcriptional efficacy of nuclear factor CREB. Nature..

[48] Handschin JC, Eppenberger U (1979). Altered cellular ratio of type I and type II cyclic AMP-dependent protein kinase in human mammary tumors. FEBS Lett.

[49] Basso F, Rocchetti F, Rodriguez S, Nesterova M, Cormier F, Stratakis CA (2014). Comparison of the effects of PRKAR1A and PRKAR2B depletion on signaling pathways, cell growth, and cell cycle control of adrenocortical cells. Horm Metab Res.

[50] Sandrini F, Kirschner LS, Bei T, Farmakidis C, Yasufuku-Takano J, Takano K (2002). PRKAR1A, one of the Carney complex genes, and its locus (17q22-24) are rarely altered in pituitary tumours outside the Carney complex. J Med Genet.

[51] Mole DR, Blancher C, Copley RR, Pollard PJ, Gleadle JM, Ragoussis J (2009). Genome-wide association of hypoxia-inducible factor (HIF)-1alpha and HIF-2alpha DNA binding with expression profiling of hypoxia-inducible transcripts. J Biol Chem.

[52] Elliott MR, Tolnay M, Tsokos GC, Kammer GM (2003). Protein kinase A regulatory subunit type II beta directly interacts with and suppresses CREB transcriptional activity in activated T cells. J Immunol.

[53] Bertout JA, Patel SA, Simon MC (2008). The impact of O_2_ availability on human cancer. Nat Rev Cancer.

[54] Semenza GL (2017). Hypoxia-inducible factors: coupling glucose metabolism and redox regulation with induction of the breast cancer stem cell phenotype. EMBO J.

[55] Carlucci A, Adornetto A, Scorziello A, Viggiano D, Foca M, Cuomo O (2008). Proteolysis of AKAP121 regulates mitochondrial activity during cellular hypoxia and brain ischaemia. EMBO J.

[56] Shaikh D, Zhou Q, Chen T, Ibe JC, Raj JU, Zhou G (2012). cAMP-dependent protein kinase is essential for hypoxia-mediated epithelial-mesenchymal transition, migration, and invasion in lung cancer cells. Cell Signal.

[57] Taylor SS, Ilouz R, Zhang P, Kornev AP (2012). Assembly of allosteric macromolecular switches: lessons from PKA. Nat Rev Mol Cell Biol.

[58] Tortora G, Pepe S, Bianco C, Baldassarre G, Budillon A, Clair T (1994). The RI alpha subunit of protein kinase A controls serum dependency and entry into cell cycle of human mammary epithelial cells. Oncogene..

[59] Tsigginou A, Bimpaki E, Nesterova M, Horvath A, Boikos S, Lyssikatos C (2012). PRKAR1A gene analysis and protein kinase A activity in endometrial tumors. Endocr Relat Cancer.

[60] Nesterova M, Yokozaki H, McDuffie E, Cho-Chung YS (1996). Overexpression of RII beta regulatory subunit of protein kinase A in human colon carcinoma cell induces growth arrest and phenotypic changes that are abolished by site-directed mutation of RII beta. Eur J Biochem.

[61] Krishnamachary B, Zagzag D, Nagasawa H, Rainey K, Okuyama H, Baek JH (2006). Hypoxia-inducible factor-1-dependent repression of E-cadherin in von Hippel-Lindau tumor suppressor-null renal cell carcinoma mediated by TCF3, ZFHX1A, and ZFHX1B. Cancer Res.

[62] Koshiji M, To KK, Hammer S, Kumamoto K, Harris AL, Modrich P (2005). HIF-1alpha induces genetic instability by transcriptionally downregulating MutSalpha expression. Mol Cell.

[63] Kadonaga JT, Carner KR, Masiarz FR, Tjian R (1987). Isolation of cDNA encoding transcription factor Sp1 and functional analysis of the DNA binding domain. Cell..

[64] Kurten RC, Levy LO, Shey J, Durica JM, Richards JS (1992). Identification and characterization of the GC-rich and cyclic adenosine 3’,5’-monophosphate (cAMP)-inducible promoter of the type II beta cAMP-dependent protein kinase regulatory subunit gene. Mol Endocrinol.

[65] Kietzmann T, Mennerich D, Dimova EY (2016). Hypoxia-inducible factors (HIFs) and phosphorylation: impact on stability, localization, and transactivity. Front Cell Dev Biol.

[66] Pullamsetti SS, Banat GA, Schmall A, Szibor M, Pomagruk D, Hänze J (2013). Phosphodiesterase-4 promotes proliferation and angiogenesis of lung cancer by crosstalk with HIF. Oncogene..

[67] Toffoli S, Feron O, Raes M, Michiels C (2007). Michiels, Intermittent hypoxia changes HIF-1alpha phosphorylation pattern in endothelial cells: unravelling of a new PKA-dependent regulation of HIF-1alpha. Biochim Biophys Acta.

[68] Bullen JW, Tchernyshyov I, Holewinski RJ, DeVine L, Wu F, Venkatraman V (2016). Protein kinase A-dependent phosphorylation stimulates the transcriptional activity of hypoxia-inducible factor 1. Sci Signal.

[69] Cheong HI, Asosingh K, Stephens OR, Queisser KA, Xu W, Willard B (2016). Hypoxia sensing through beta-adrenergic receptors. JCI Insight.

[70] Taylor SS, Yang J, Wu J, Haste NM, Radzio-Andzelm E, Anand G (2004). PKA: a portrait of protein kinase dynamics. Biochim Biophys Acta.

[71] Skalhegg BS, Tasken K (2000). Specificity in the cAMP/PKA signaling pathway. Differential expression,regulation, and subcellular localization of subunits of PKA. Front Biosci.

