# Supplementary material for: Hypoxia and the hypoxia inducible factor 1α activate protein kinase A by repressing RII beta subunit transcription
Source: Oncogene. 2020 Feb 28;39(16):3367–80. doi: 10.1038/s41388-020-1223-6 (PMC7160059; doi:10.1038/s41388-020-1223-6)
Supplement: Supplementary file 1 — Supplementary figures [file 41388_2020_1223_MOESM1_ESM.docx]

**Supplementary Figure 1**


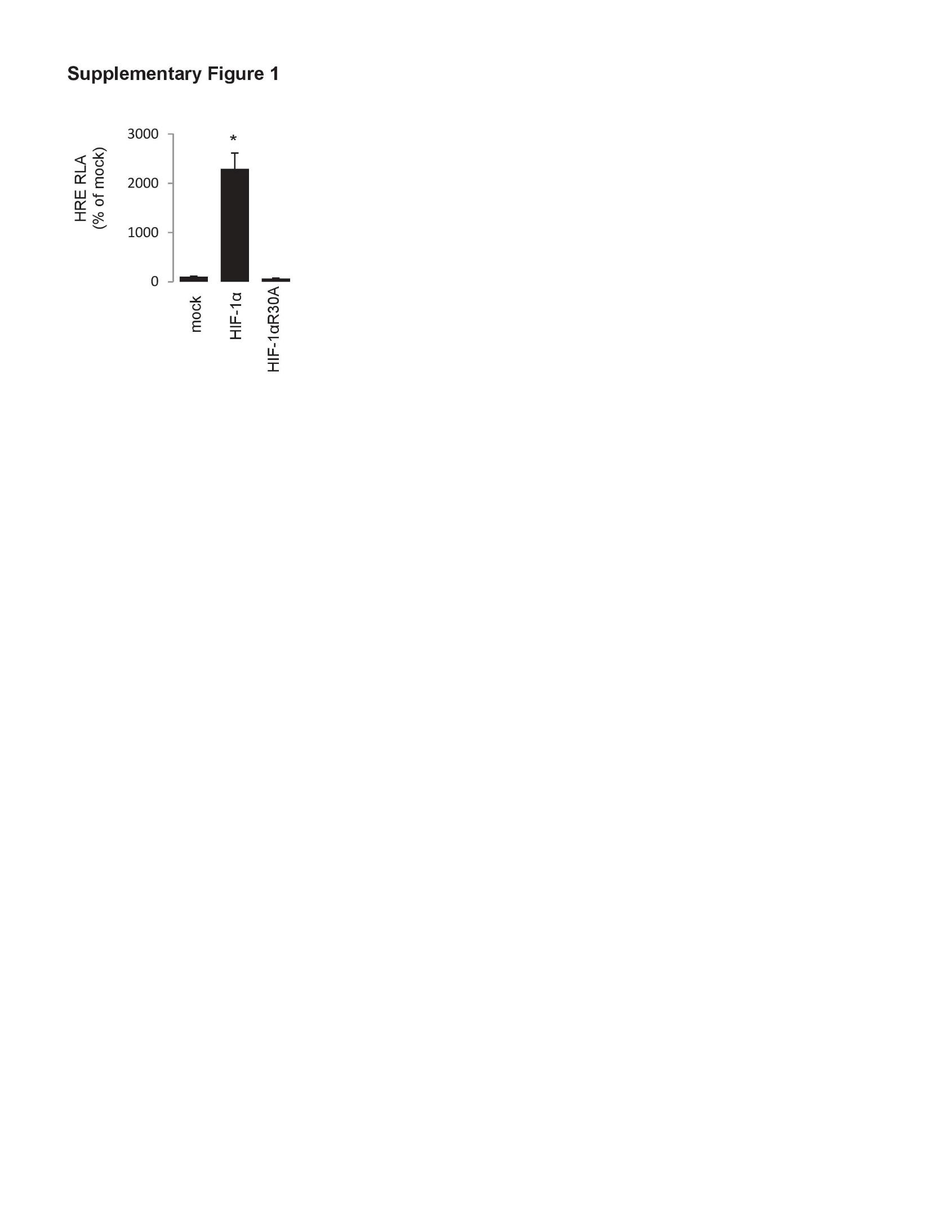


**Fig. S1.** Control experiment demonstrating loss of transcriptional activity in the HIF-1α^R30A^ mutant. The wild type HIF-1α has a dramatic effect in HRE activity. HRE, hypoxia responsive element. RLA, relative luciferase activity. Data are Luc/βGal presented as % of mock (pCMV empty plasmid) control. **P*<0.001 to mock (Student’s *t*-test).

**Supplementary Figure 2**


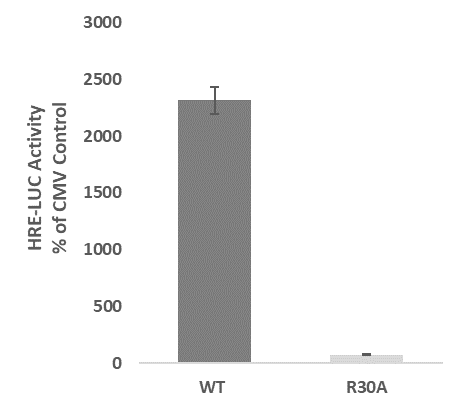


*

**Fig. S2.** Wild-Type HIF-1a (WT) activates HRE-driven transcription as measured by luciferase activity in GH3 cells transfected with a HIF-1αR30A versus wild-type HIF-1α plasmids. Data are means±SEM from 3 experiments and are presented as percentage of mock transfected controls. **P*<0.05 to mock (Student’s *t*-test).

|  | **Mock** | | | | **HIF-1a** | | | |
| --- | --- | --- | --- | --- | --- | --- | --- | --- |
| **hours** | **0** | **1** | **3** | **6** | **0** | **1** | **3** | **6** |
| **pCREB/Total CREB** | 0,00 | 0,85 | 1,00 | 0,00 | 0,97 | 1,25 | 1,09 | 0,96 |

**Supplementary Figure 3**

**Fig. S3.** Densitometric quantification of western blot bands performed in ImageJ. Values are expressed as ratio of phospho-CREB/Total CREB signal intensity.

**Supplementary Figure 4**

**
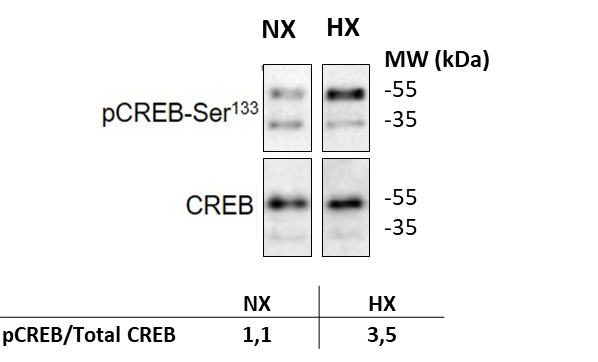
**

**Fig. S4.** Hypoxia increases phospho-CREB expression in GH3 cells

GH3 cells harvested after 3h of hypoxic incubation versus normoxic control cells show an increase of pCREB protein expression as measured by western blot and quantified as pCREB/Total CREB using the ImageJ software tool.

**Supplementary Figure 5**


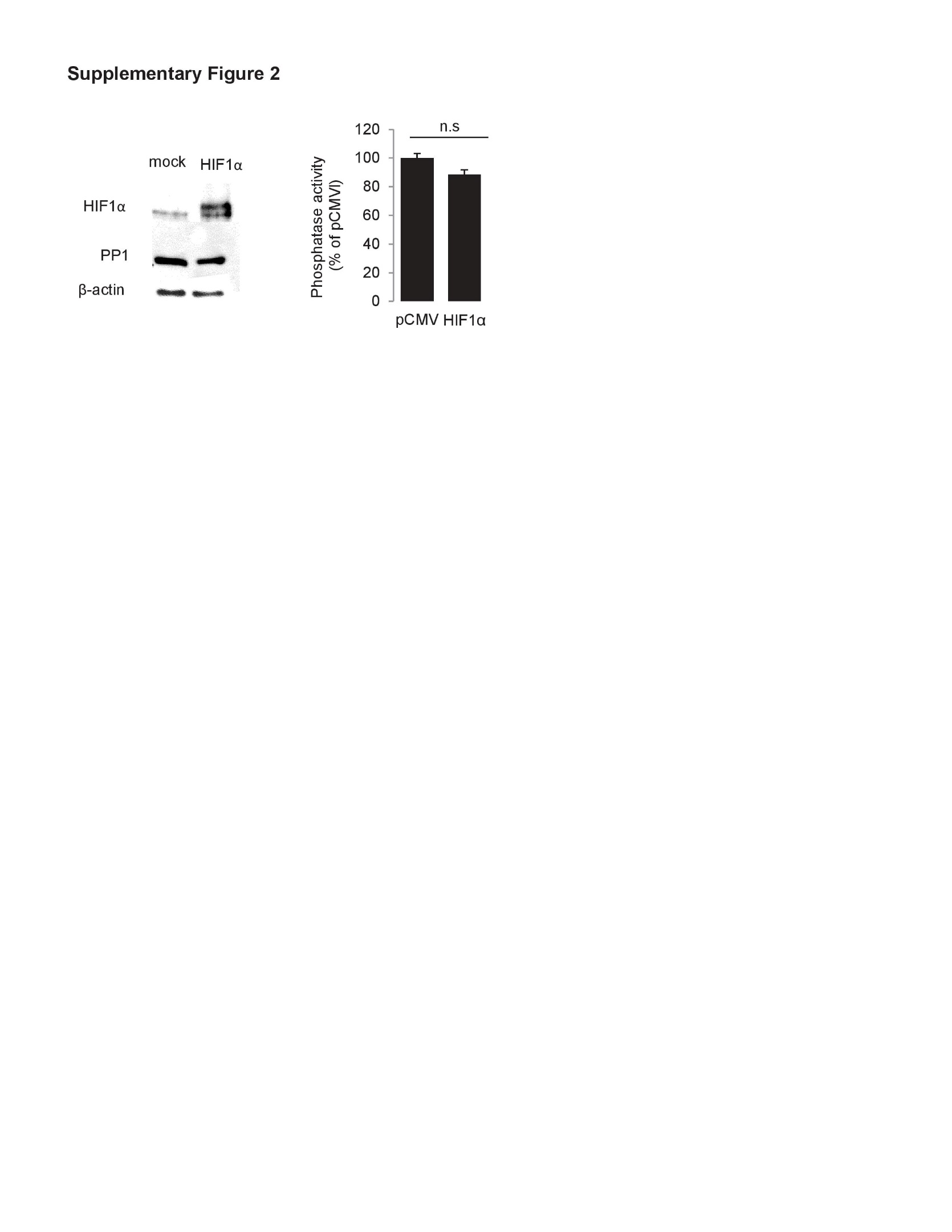


**Fig. S5.** HIF-1a does not affect PP1 protein levels as determined by western blot and phosphatase activity. Data are arbitrary units normalized to input protein and representative of 2 experiments performed in triplicate. **P*<0.05 to mock (Student’s *t*-test).

**Supplementary Figure 6**

*****

**A.**

**B.**

**Fig. S6. A.** the transcriptionally inactive CREB M1 Plasmid (S133A) does not bind and activate the CRE-LUC promotor construct compared to the CREB WT plasmid (Data are means±SEM from 2 experiments and are presented as absolute values (RLU). **P*<0.05 to mock (Student’s *t*-test). **B.** transfection of the HIF-1a expression plasmid shows increase of rGH secretion in WT CREB expressing GH3 cells. This effect is abrogated by GH3 cells expressing the transcriptionally inactive CREB M1 plasmid. Data are means±SEM from 2 experiments and are presented as percent of Mock. **P*<0.05 to mock (Student’s *t*-test).

**Supplementary Figure 7**


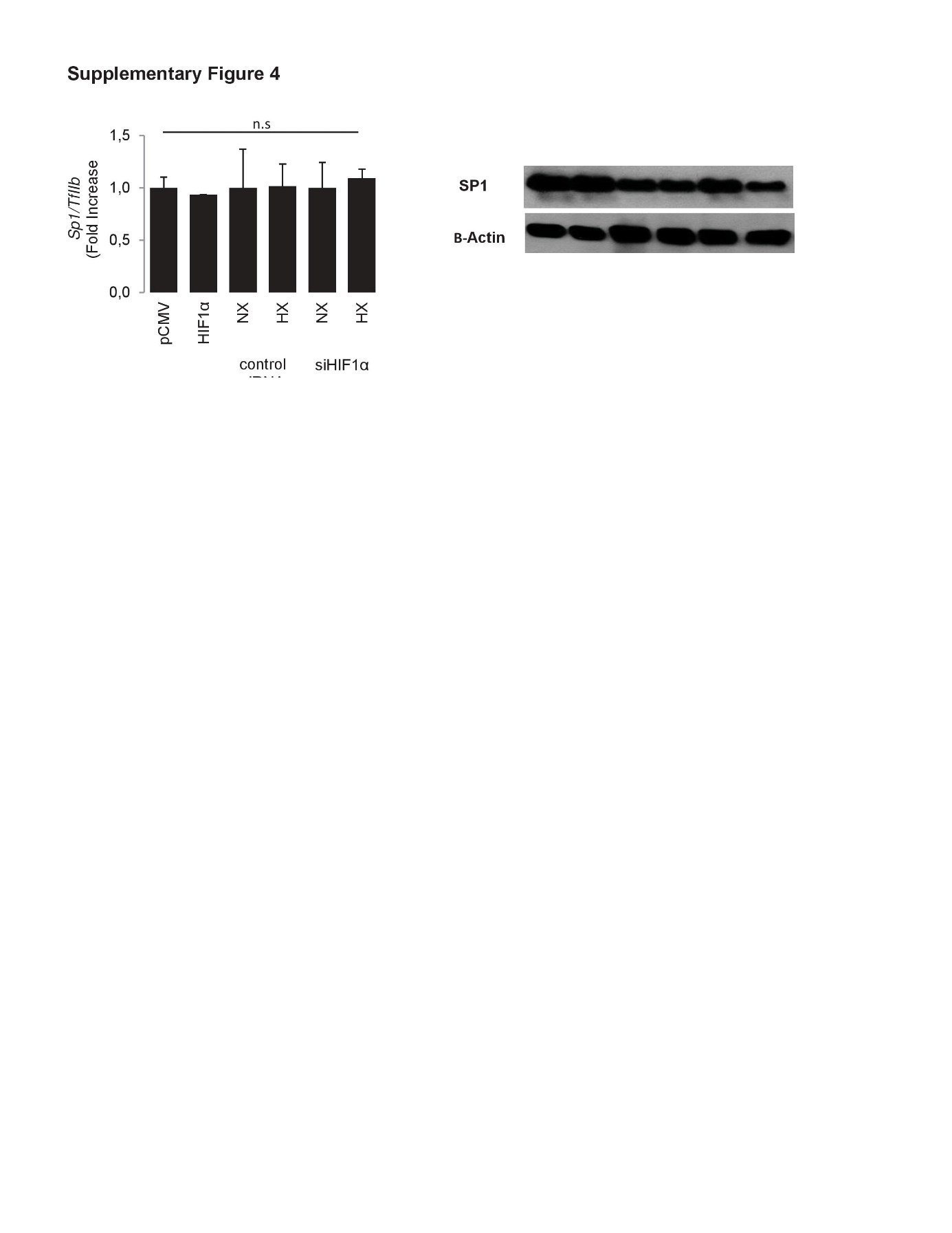


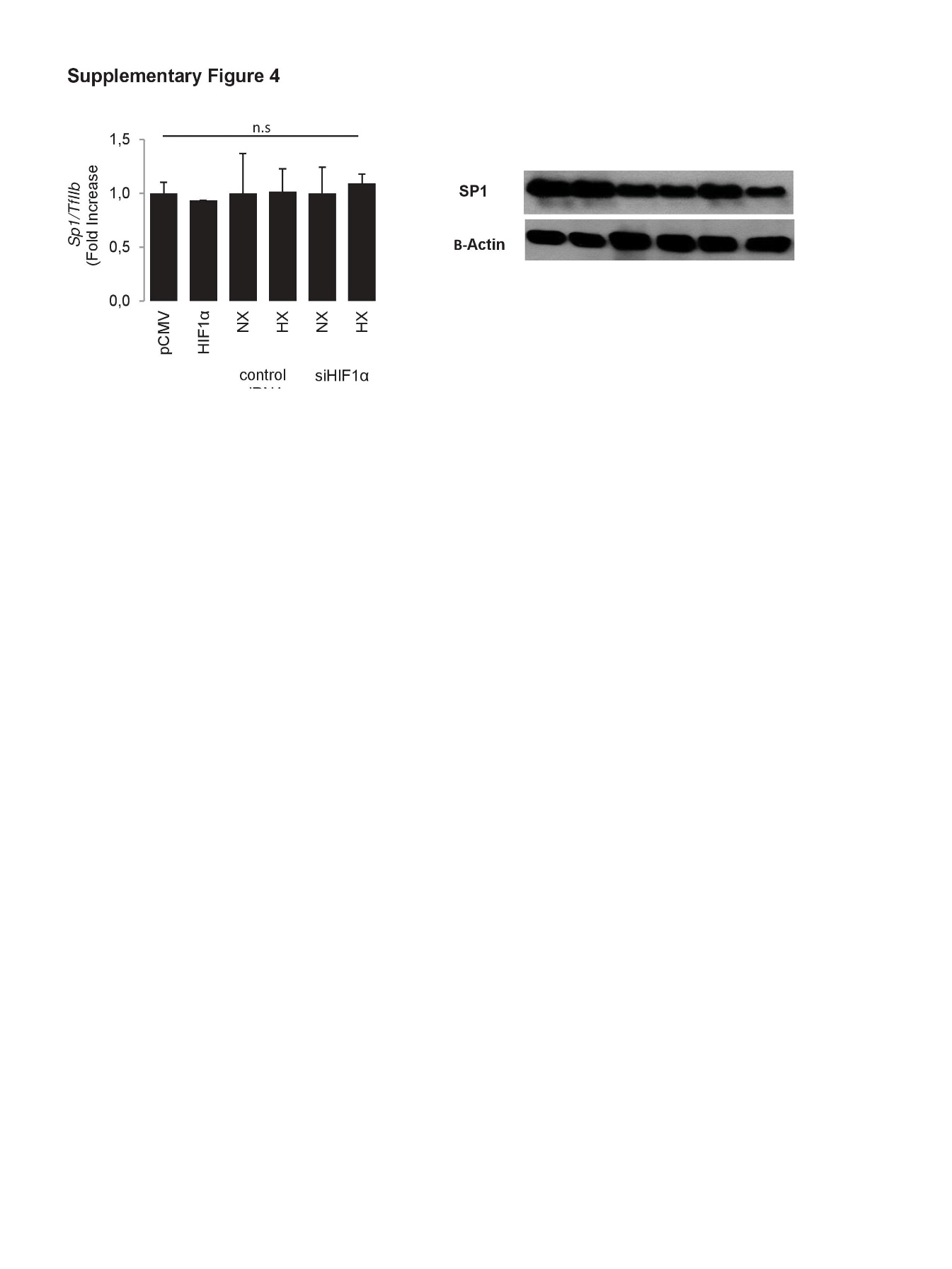


**Fig. S7.** Neither HIF-1α not hypoxia affects Sp1 transcription or protein levels as determined by real time RT-PCR and western blot. Data are representative of 2 experiments. **P*<0.05 to control (pCMV or NX) (U-Test).

**Supplementary Figure 8**


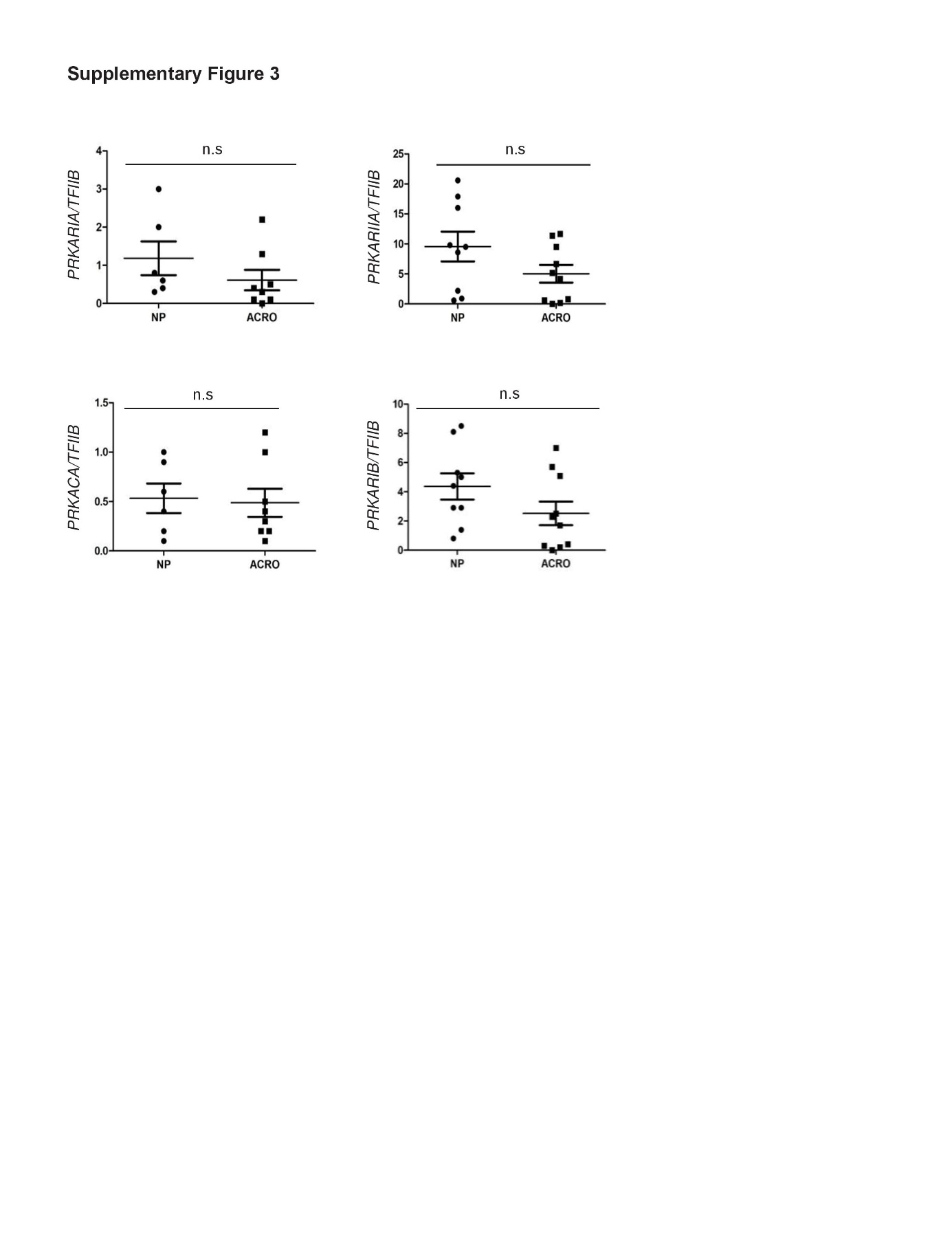


**Fig. S8.** Expression levels of the PKA regulatory and catalytic subunits on the same cohort of normal pituitaries (NP) and acromegalic tumors (ACRO) as in Figure 5c, as determined by real time RT-PCR. Data are *<gene>/TFIIB*. **P*<0.05 to NP (U-Test).
